# Response of circulating metabolites to an oral glucose challenge and risk of cardiovascular disease and mortality in the community

**DOI:** 10.1186/s12933-022-01647-w

**Published:** 2022-10-15

**Authors:** Daniel Gonzalez Izundegui, Patricia E. Miller, Ravi V. Shah, Clary B. Clish, Maura E. Walker, Gary F. Mitchell, Robert E. Gerszten, Martin G. Larson, Ramachandran S. Vasan, Matthew Nayor

**Affiliations:** 1 Department of Medicine, Boston University School of Medicine, Boston, MA USA; 2 Department of Biostatistics, Boston University School of Public Health, Boston, MA USA; 3 Vanderbilt Translational and Clinical Research Center, Cardiology Division, Vanderbilt University Medical Center, Nashville, TN USA; 4 Broad Institute of MIT and Harvard, Cambridge, MA USA; 5 Section of Preventive Medicine and Epidemiology, Department of Medicine, Boston University School of Medicine, 72 E Concord Street, Suite L-516, Boston, MA 02118 USA; 6 Department of Health Sciences, Program in Nutrition, Sargent College of Health and Rehabilitation Sciences, Boston University, Boston, MA USA; 7 Boston University’s and National Heart, Lung, and Blood Institute’s Framingham Heart Study, Framingham, MA USA; 8 Cardiovascular Engineering, Inc, Norwood, MA USA; 9 Division of Cardiovascular Medicine, Beth Israel Deaconess Medical Center, Boston, MA USA; 10 Section of Cardiovascular Medicine, Department of Medicine, Boston University School of Medicine, 72 E Concord Street, Suite L-516, Boston, MA 02118 USA; 11 Department of Epidemiology, Boston University Schools of Medicine and Public Health, Center for Computing and Data Sciences, Boston University, Boston, MA USA

**Keywords:** Metabolism, Metabolomics, Prevention, Cardiovascular disease

## Abstract

**Background:**

New biomarkers to identify cardiovascular disease (CVD) risk earlier in its course are needed to enable targeted approaches for primordial prevention. We evaluated whether intraindividual changes in blood metabolites in response to an oral glucose tolerance test (OGTT) may provide incremental information regarding the risk of future CVD and mortality in the community.

**Methods:**

An OGTT (75 g glucose) was administered to a subsample of Framingham Heart Study participants free from diabetes (n = 361). Profiling of 211 plasma metabolites was performed from blood samples drawn before and 2 h after OGTT. The log2(post/pre) metabolite levels (Δmetabolites) were related to incident CVD and mortality in Cox regression models adjusted for age, sex, baseline metabolite level, systolic blood pressure, hypertension treatment, body mass index, smoking, and total/high-density lipoprotein cholesterol. Select metabolites were related to subclinical cardiometabolic phenotypes using Spearman correlations adjusted for age, sex, and fasting metabolite level.

**Results:**

Our sample included 42% women, with a mean age of 56 ± 9 years and a body mass index of 30.2 ± 5.3 kg/m^2^. The pre- to post-OGTT changes (Δmetabolite) were non-zero for 168 metabolites (at FDR ≤ 5%). A total of 132 CVD events and 144 deaths occurred during median follow-up of 24.9 years. In Cox models adjusted for clinical risk factors, four Δmetabolites were associated with incident CVD (higher glutamate and deoxycholate, lower inosine and lysophosphatidylcholine 18:2) and six Δmetabolites (higher hydroxyphenylacetate, triacylglycerol 56:5, alpha-ketogluturate, and lower phosphatidylcholine 32:0, glucuronate, N-monomethyl-arginine) were associated with death (P < 0.05). Notably, baseline metabolite levels were not associated with either outcome in models excluding Δmetabolites. The Δmetabolites exhibited varying cross-sectional correlation with subclinical risk factors such as visceral adiposity, insulin resistance, and vascular stiffness, but overall relations were modest. Significant Δmetabolites included those with established roles in cardiometabolic disease (e.g., glutamate, alpha-ketoglutarate) and metabolites with less defined roles (e.g., glucuronate, lipid species).

**Conclusions:**

Dynamic changes in metabolite levels with an OGTT are associated with incident CVD and mortality and have potential relevance for identifying CVD risk earlier in its development and for discovering new potential therapeutic targets.

**Supplementary Information:**

The online version contains supplementary material available at 10.1186/s12933-022-01647-w.

## Introduction

Traditional cardiovascular disease (CVD) risk factors are highly prevalent in the general population and account for a significant proportion of attributable risk [1, 2]. Despite progress over recent decades in CVD risk prediction and in treating clinical risk factors to prevent CVD events (primary prevention) [3], the global burden of CVD remains unacceptably high [4]. This high residual burden is attributable partly to the enormous challenge of reducing clinical risk factors to optimal levels in the general population [5]. Additionally, even when optimal levels of a risk factor are achieved with medications, CVD risk often remains higher when compared to those who never developed the risk factors [6, 7]. Moreover, a substantial proportion of CVD events occur in individuals who are not predicted to be at elevated risk based on traditional risk factor thresholds [8]. Therefore, it is necessary to identify novel biomarkers that are evident earlier in the course of risk factor pathogenesis (primordial prevention) to augment clinical risk assessment, facilitate lifestyle interventions at earlier—and more modifiable [9]—stages of development of disease propensity, and identify new potential therapeutic targets.

**Fig. 1:**
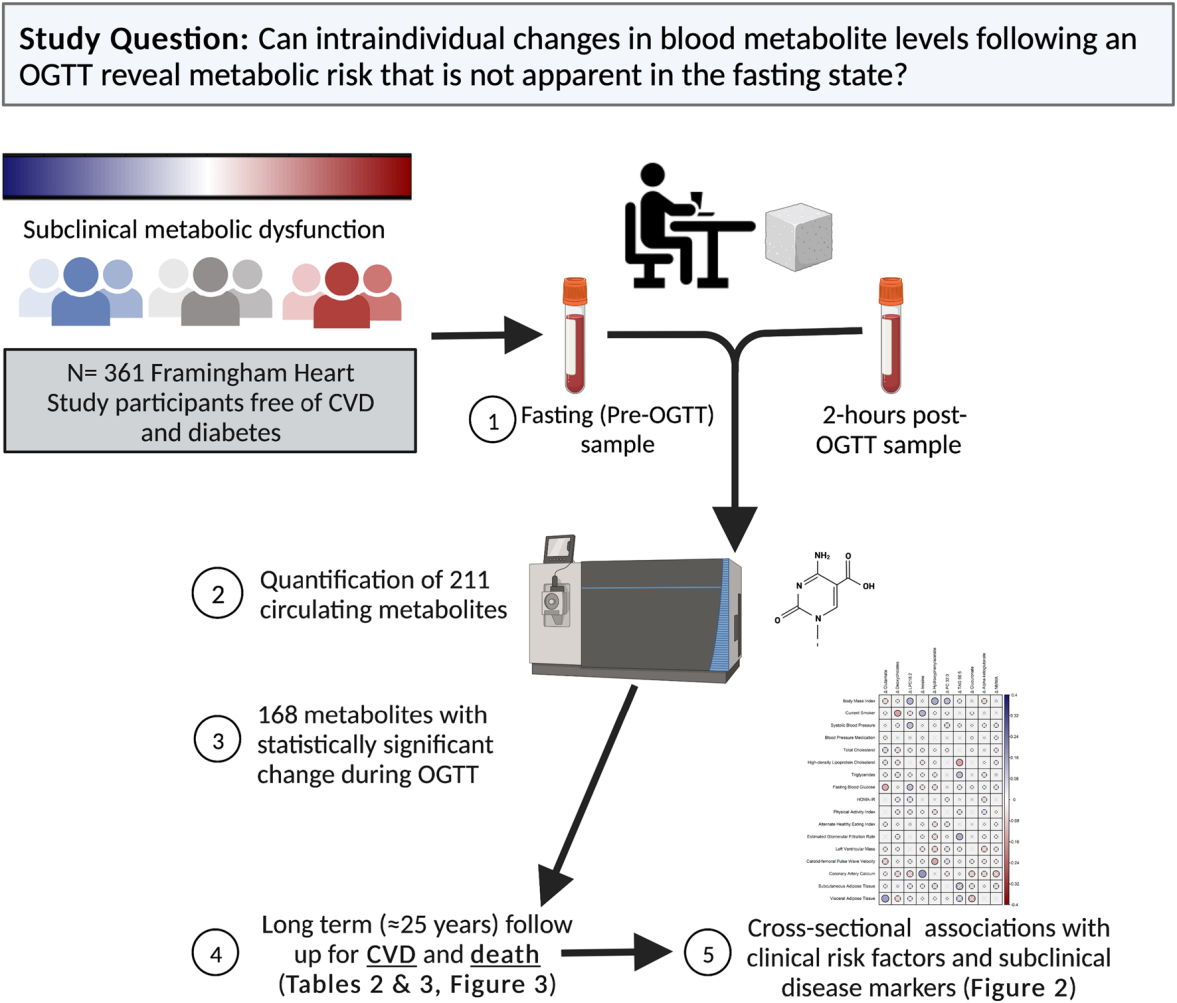
Schematic of the study design

Circulating metabolites are a valuable resource for the discovery of biomarkers of early disease risk as they provide a dynamic snapshot of diverse metabolic functions. Accordingly, fasting metabolite levels have been linked to important CVD-related outcomes such as diabetes [10–13], obesity [14, 15], hypertension [16], CVD [17, 18], longevity [19], and mortality [20]. However, these prior studies are limited by a reliance on traditional risk factors to define metabolite profiles and by biomarker assessment at a single time point. As metabolites change dynamically in response to physiological conditions [21], they might also prove useful for characterizing interindividual differences in the metabolic response to stress (perturbation), an emerging indicator of physiological health [22, 23]. Indeed, widespread changes in the circulating metabolome have been reported in response to the acute metabolic stress of an oral glucose tolerance test (OGTT), with differences in these changes in individuals with versus without insulin resistance [24]. However, it is currently unknown whether such interindividual variability in metabolite changes after an OGTT might provide information on the long-term risk of CVD or premature mortality. To address this research question, blood metabolites were quantified before and 2 h after an OGTT in well-phenotyped, nondiabetic, community-dwelling participants of the Framingham Heart Study (FHS) with > 20 years of longitudinal follow-up for CVD events and mortality. Our overall objective was to test the hypothesis that intraindividual changes in blood metabolite levels following an OGTT can uncover interindividual variation in metabolic risk that is not apparent in the fasting state (Fig. 1).

## Methods

### Study sample

The FHS Offspring cohort is an observational, prospective, community-based cohort initially recruited in 1971 and followed with serial examinations subsequently [25]. At their 5th examination cycle (1991–1995), a subsample of this cohort without diabetes underwent an OGTT with blood sampling for metabolite profiling pre-OGTT and 2 h post [24]. This subsample included 189 individuals who subsequently developed diabetes and 189 propensity-matched controls who did not develop diabetes, as described previously [10]. For the present investigation, we included 361 individuals with metabolite profiling performed and who were free of CVD at the baseline (5th) examination cycle. The study was approved by the Institutional Review Board at Boston University Medical Campus/Boston Medical Center and all participants provided written informed consent.

### OGTT protocol and metabolite profiling

Participants presented after a ≥ 8 h fast. Blood samples were drawn before and 2 hafter a 75 g OGTT. Samples were centrifuged immediately and stored at − 80 °C until assayed. Plasma metabolites were analyzed using liquid chromatography–mass spectrometry (LC–MS) methods at the Broad Institute of Harvard and Massachusetts Institute of Technology (Cambridge, MA) in 2008–2011, as described [10, 24]. Briefly, metabolites were extracted with 75% acetonitrile/25% methanol for positively charged polar compounds and 80% methanol for negatively charged polar compounds. Samples were centrifuged (10 min, 10,000 rpm, 4 °C) and supernatants were injected directly. LC–MS data were acquired using a 4000 QTRAP triple quadrupole mass spectrometer (Applied Biosystems/Sciex, Foster City, CA) and a multiplexed LC system comprised of two 1200 Series pumps (Agilent Technologies, Santa Clara, CA). Polar plasma metabolites were measured using hydrophilic interaction chromatography and tandem MS with electrospray ionization and multiple reaction monitoring scans in the positive ion mode. Complementary analysis of small molecules ionized in the negative mode were also assayed. After excluding drug metabolites and metabolites with more than 25% missingness, 211 metabolites were available for analysis.

### Covariate and outcome assessment

Diabetes was defined as a fasting blood glucose ≥ 126 mg/dL, nonfasting glucose ≥ 200 mg/dL, or the use of blood sugar-lowering medications. Systolic blood pressure was measured by manual mercury column sphygmomanometer on seated participants and the average of two readings was recorded. Smoking status (in the year preceding the FHS examination) was assessed by self-report. Dietary quality and physical activity were assessed by questionnaire and expressed as the Alternative Healthy Eating Index-2010 and physical activity index [26, 27]. Homeostatic Model of Insulin Resistance (HOMA-IR) was calculated as fasting glucose (nmol/L) * fasting insulin (µU/ml)/22.5. The estimated glomerular filtration rate was calculated using the Chronic Kidney Disease Epidemiology Collaboration Equation. [28]. Left ventricular mass was assessed by a transthoracic echocardiogram and calculated using the Devereux formula [29]. At the 7th examination cycle (1998–2001), carotid-femoral pulse wave velocity (CFPWV; analyzed as −1000/CFPWV), coronary artery calcium (analyzed as natural log[1 + coronary artery calcium score]), and subcutaneous and visceral adipose tissue volume were assessed using standardized protocols [30–32]. FHS participants are under surveillance for the development of CVD events, which are adjudicated during a consensus review of pertinent medical records by three investigators. For the present investigation, incident CVD events were defined as fatal or nonfatal myocardial infarction, stroke, intermittent claudication, or heart failure using standardized criteria [33].

### Statistical analysis

First, we compared pre- and post-OGTT log2(metabolite levels) using paired *t*-tests. For each metabolite with changes from pre- to post-OGTT at a false discovery rate (FDR) of ≤ 5%, we calculated the log2 fold change (log2[post/pre]) and fold-changes were standardized to mean = 0, standard deviation = 1. Metabolite fold-changes were then related to incident CVD and mortality in multivariable-adjusted Cox proportional hazards regression models. Models were initially adjusted for age, sex, and baseline (fasting) metabolite level and were then additionally adjusted for traditional CVD risk factors used for clinical risk prediction: systolic blood pressure, hypertension treatment status, body mass index, smoking status, and total/HDL cholesterol [34]. In separate models, we evaluated the associations of the fasting metabolite levels with CVD and death to facilitate comparison. We conducted sensitivity analyses in which metabolites associated with CVD or mortality were additionally adjusted for fasting blood glucose and HOMA-IR. Hazard ratios (HRs) were calculated by taking the exponential of the regression coefficient (*exp(β)*) from the corresponding Cox proportional hazard model along with a 95% confidence interval (CI). In exploratory analyses, we evaluated the relations of the metabolite fold-changes with clinical risk factors and subclinical CVD markers using partial correlations (Spearman) adjusted for age, sex, and fasting metabolite level. Risk factors and subclinical CVD markers included BMI, smoking, systolic blood pressure, blood pressure medication use, total cholesterol, high-density lipoprotein cholesterol, triglycerides, fasting blood glucose, HOMA-IR, physical activity index, alternate health eating index, estimated glomerular filtration rate, left ventricular mass, carotid-femoral pulse wave velocity, coronary artery calcium, subcutaneous and visceral adipose tissue density. A 2-sided P < 0.05 was used to determine the statistical significance without adjustment for multiple testing. Analyses were conducted using SAS version 9.4 (Cary, NC) and with R statistical software, version 4.03 (Foundation for Statistical Computing, Vienna, Austria).

## Results

### Study sample characteristics

Our analytic subsample consisted of 361 individuals (mean age 56 ± 9 years) with 151 (42%) women, and a mean body mass index in the obese range (30.2 ± 5.3 kg/m^2^), Table 1 and Additional file 1: Table S1. Compared with the larger FHS Generation 2 cohort, this subsample had similar age, with a lower proportion of women and modestly higher levels of cardiovascular risk factors (Additional file 1: Table S2).

**Table 1:** Characteristics of the study sample

Characteristic	Study sample (N = 361)
Age, years	56 ± 9
Women	151 (42%)
Body mass index, kg/m^2^	30.2 ± 5.3
Systolic blood pressure, mm Hg	133 ± 18
Hypertension treatment	103 (29%)
Current smoking	58 (16%)
Total cholesterol, mg/dl	210.8 ± 36.3
HDL cholesterol, mg/dl	45.3 ± 12.9
Triglycerides, mg/dl	170 ± 105
Fasting blood glucose, mg/dl	105 ± 9
HOMA-IR	1.30 ± 0.52
Physical activity index	35 ± 8
Alternate healthy eating index	52 ± 12
Estimated glomerular filtration rate, ml/min/1.73 m^2^	89 ± 19
Left ventricular mass, grams	177 ± 40
Carotid femoral pulse wave velocity, m/s	10.4 (8.6–12.2)
Coronary artery calcium, Hounsfield units	147 (12–568)
Subcutaneous adipose tissue, cm^3^	3209 ± 1337
Visceral adipose tissue, cm^3^	2852 ± 1104

Sample sizes for variables not available in all participants: HOMA-IR, N = 359; physical activity index, N = 352; alternate healthy eating index, N = 331; estimated glomerular filtration rate, N = 319; left ventricular mass, N = 253; carotid-femoral pulse wave velocity, N = 234; coronary artery calcium, N = 131; subcutaneous adipose tissue, N = 138; visceral adipose tissue, N = 138

Data in the table are mean ± SD, median (25th–75th percentile), or N(% of total)

### Association of pre- to post-OGTT metabolite changes with incident CVD

Of the 211 assayed metabolites, changes from pre- to post-OGTT were observed for 168 metabolites (80%) at FDR ≤ 5% (Additional file 1: Table S3). During a median follow-up of 24.9 (limits 7.5–28.3) years, a first CVD event occurred in 132 individuals. In Cox models adjusted for age, sex, and fasting metabolite level, the pre- to post-OGTT change in the levels (Δmetabolite) of 13 metabolites were associated with incident CVD (Table 2). A nominal significance threshold of P < 0.05 was used for all prospective analyses. This included several triacylglycerol (TAG) species, metabolites with putative links with cardiometabolic disease (e.g., glutamate, lactate, isoleucine, alanine [35]), and metabolites with less well-established roles in cardiometabolic disease (e.g., gentisate, cholesterol ester 20:5), Table 2. After additional adjustment for traditional CVD risk factors, the association of four Δmetabolites and incident CVD remained statistically significant: higher glutamate and deoxycholate and lower lysophosphatidylcholine (LPC)18:2 and inosine (Table 2). Notably, in models not accounting for the post-OGTT change, six metabolites were associated with incident CVD in age- and sex-adjusted models (exhibiting directional concordance with the Δmetabolite analyses), but none of the fasting metabolite levels were associated with incident CVD in models adjusted for traditional CVD risk factors in our sample (Table 2).

**Table 2:** Association of Δmetabolites with incident CVD

	Δ metabolites				Fasting metabolites			
	Model 1		Model 2		Model 1		Model 2	
Metabolite	HR (95% CI)	P-value	HR (95% CI)	P-value	HR (95% CI)	P-value	HR (95% CI)	P-value
Glutamate	**1.22 (1.02–1.47)**	**0.033**	**1.24 (1.03–1.5)**	**0.026**	1.15 (0.96–1.37)	0.13	1.13 (0.93–1.36)	0.22
Deoxycholate	1.13 (0.93–1.37)	0.21	**1.26 (1.02–1.55)**	**0.030**	1.02 (0.84–1.23)	0.84	1.05 (0.87–1.27)	0.61
LPC 18:2	0.82 (0.67–1.00)	0.056	**0.80 (0.65–0.99)**	**0.037**	**0.79 (0.63–0.99)**	**0.041**	0.84 (0.66–1.07)	0.15
Inosine	0.93 (0.77–1.13)	0.47	**0.82 (0.67–0.99)**	**0.043**	1.07 (0.88–1.29)	0.49	0.97 (0.80–1.18)	0.78
TAG 58:12	**1.24 (1.03–1.49)**	**0.023**	1.20 (1.00–1.43)	0.052	**1.22 (1.01–1.46)**	**0.034**	1.17 (0.96–1.42)	0.12
TAG 50:2	**1.32 (1.08–1.61)**	**0.007**	1.22 (0.99–1.51)	0.07	**1.30 (1.08–1.57)**	**0.005**	1.11 (0.90–1.36)	0.33
Lactate	**0.81 (0.67–0.97)**	**0.026**	0.84 (0.69–1.02)	0.08	1.11 (0.93–1.34)	0.25	1.10 (0.91–1.33)	0.32
Isoleucine	**1.20 (1.00–1.43)**	**0.046**	1.16 (0.97–1.38)	0.12	1.06 (0.88–1.28)	0.55	0.96 (0.78–1.18)	0.69
Cholesterol ester 20:5	**0.82 (0.68–0.98)**	**0.032**	0.87 (0.72–1.04)	0.13	0.93 (0.78–1.10)	0.39	1.00 (0.83–1.22)	0.97
TAG 48:1	**1.25 (1.04–1.51)**	**0.018**	1.17 (0.96–1.43)	0.13	**1.24 (1.03–1.49)**	**0.022**	1.06 (0.87–1.30)	0.55
TAG 50:3	**1.27 (1.04–1.54)**	**0.017**	1.16 (0.95–1.43)	0.14	**1.24 (1.03–1.50)**	**0.025**	1.05 (0.84–1.32)	0.67
Alanine	**0.80 (0.66–0.97)**	**0.024**	0.86 (0.70–1.05)	0.15	1.00 (0.83–1.21)	0.97	0.95 (0.78–1.16)	0.61
Aminoisobutyric	**1.20 (1.00–1.43)**	**0.044**	1.13 (0.94–1.36)	0.18	0.97 (0.81–1.16)	0.74	1.04 (0.87–1.25)	0.64
Gentisate	**0.82 (0.68–0.99)**	**0.038**	0.90 (0.74–1.09)	0.27	0.85 (0.70–1.04)	0.11	0.83 (0.68–1.01)	0.06
TAG 54:3	**1.21 (1.01–1.45)**	**0.040**	1.11 (0.91–1.34)	0.29	1.18 (0.99–1.41)	0.07	1.01 (0.82–1.25)	0.92
TAG 48:2	**1.21 (1.00–1.45)**	**0.046**	1.11 (0.91–1.35)	0.32	**1.21 (1.01–1.45)**	**0.041**	1.03 (0.83–1.28)	0.78

Δ Metabolite is the log2 fold-change from pre- to post-OGTT and baseline metabolites were log-transformed. Baseline and change metabolite values were standardized (mean 0 and SD 1)

The hazard ratio (HR) represents the relative hazard for a 1-SD higher log2 fold-change in the metabolite

P-values are not adjusted for multiple hypothesis testing. Values in bold represent statistically significant associations at a P < 0.05 level

The Δ metabolite models are also adjusted for fasting metabolite levels

Model 1 is adjusted for age and sex

Model 2 is adjusted also for BMI, smoking, hypertension treatment, systolic blood pressure, and total/HDL cholesterol

### Association of pre- to post-OGTT metabolite changes with incident death

A total of 144 deaths occurred during the follow up period. In Cox models adjusted for age, sex, and fasting metabolite levels, five Δmetabolites were associated with mortality (p < 0.05, Table 3): hydroyphenylacetate, TAG 56:5, glucuronate, sucrose, and propionate. After additional adjustment for traditional clinical risk factors, six Δmetabolites were associated with mortality (Table 3). These included lipid species such as TAG 56:5 and phosphatidylcholine 32:0, as well as the phenol hydroxyphenylacetate (previously implicated in unhealthy aging[36, 37]), nitric oxide inhibitor N-monomethyl arginine (NMMA), and ⍺-ketoglutarate, which promotes longevity in animal models [38] (Table 4). Fasting metabolite levels were not statistically significantly associated with death in models that did not account for changes in metabolites in response to the OGTT (Table 3).

**Table 3:** Association of Δmetabolites with mortality

Metabolite	Δ metabolites				Fasting metabolites			
	Model 1		Model 2		Model 1		Model 2	
	HR (95% CI)	P-value	HR (95% CI)	P-value	HR (95% CI)	P-value	HR (95% CI)	P-value
Hydroxyphenylacetate	**1.29 (1.07–1.56)**	**0.007**	**1.29 (1.06–1.57)**	**0.010**	0.98 (0.82–1.17)	0.82	0.96 (0.80–1.15)	0.64
PC 32:0	0.86 (0.73–1.00)	0.053	**0.81 (0.69–0.95)**	**0.011**	1.10 (0.91–1.32)	0.31	1.05 (0.87–1.27)	0.59
TAG 56:5	**1.26 (1.04–1.53)**	**0.02**	**1.27 (1.03–1.55)**	**0.023**	1.03 (0.86–1.23)	0.73	0.95 (0.79–1.14)	0.56
Glucuronate	**0.85 (0.74–0.99)**	**0.033**	**0.84 (0.72–0.98)**	**0.028**	0.99 (0.83–1.18)	0.87	0.95 (0.78–1.14)	0.56
⍺-ketoglutarate	1.21 (1.00–1.46)	0.052	**1.22 (1.01–1.46)**	**0.037**	1.12 (0.92–1.37)	0.24	1.06 (0.86–1.30)	0.61
NMMA	0.86 (0.72–1.02)	0.09	**0.83 (0.69–1.00)**	**0.045**	1.11 (0.93–1.33)	0.26	1.10 (0.91–1.32)	0.33
Sucrose	**1.22 (1.01–1.46)**	**0.037**	1.21 (0.99–1.47)	0.058	0.95 (0.76–1.19)	0.67	0.88 (0.70–1.11)	0.27
Propionate	**1.27 (1.02–1.59)**	**0.032**	1.25 (0.99–1.59)	0.06	0.97 (0.76–1.24)	0.80	0.99 (0.77–1.27)	0.92

Δ Metabolite is the log2 fold-change from pre- to post-OGTT and baseline metabolites were log-transformed. Baseline and change metabolite values were standardized (mean 0 and SD 1)

The hazard ratio (HR) represents the relative hazard for a 1-SD higher log2 fold-change in the metabolite. P-values are not adjusted for multiple hypothesis testing. Values in bold represent statistically significant associations at a P < 0.05 level

The Δ metabolite models are also adjusted for fasting metabolite levels

Model 1 is adjusted for age and sex

Model 2 is adjusted also for BMI, smoking, hypertension treatment, systolic blood pressure, and total/HDL cholesterol

**Table 4:** Functional significance of select metabolites

Metabolite	Biological pathway/function	Direction of change with OGTT (fold change) (%)	Direction of association of change after OGTT with		Biological functions and previous association with CVD and cardiometabolic disease
			CVD	Mortality	
Glutamate	Glutamate/glutamine cycle	↓ (20)	↑		Contributes to gluconeogenesis, proteolysis, inflammation [52, 53], cellular metabolism (anaplerosis); high fasting glutamate associated with higher CVD and diabetes risk [39, 40]
Inosine	Nucleoside	↓ (58)	↓		Intermediate in purine biosynthesis and secondary metabolite of purine degradation (from hypoxanthine); fasting levels higher in diabetes, but theorized to have cardioprotective effects [56, 57]
Deoxycholate	Bile acid	↓ (28)	↑		Product of cholesterol metabolism; higher fasting levels linked with diabetes risk [54]; total bile acids associated with CVD [58]; pro-inflammatory [59]
Phosphatidylcholine (PC) 32:0	Glycerophospholipid	↓ (4)		↓	Structural role in cell membranes; reservoir for circulating fatty acids [60]; several PCs have been associated (both directly and inversely) with CVD with direct mechanisms unknown [61]
LysoPC 18:2	Glycerophospholipid	↓ (4)	↓		Produced from partial hydrolysis of PCs; bioactive lipid involved in monocyte recruitment, vascular smooth muscle proliferation, endothelial dysfunction; fasting levels associated with lower risk of atherosclerosis [60, 62]
Hydroxy phenylacetate	Phenol	↓ (18)		↑	Derived from acetate metabolism; implicated in CVD risk and unhealthy aging [36, 37], affected by microbial metabolism [63]
Triacylglycerol (TAG) 56:5	Triacylglycerol	↑ (8)		↑	TAG species demonstrate different associations with cardiometabolic disease; lower carbon number and double bond content associated with insulin resistance and higher diabetes risk [13]; uptake from blood stimulated by the liver so increased circulating after a meal may partially reflect insulin resistance [64]
Glucuronate	Vitamin C precursor	↓ (10)		↓	Derived from glucose, aids in the elimination of toxins; plasma levels have been previously related to reduced longevity [65]
⍺-Ketoglutarate	Tricarboxylic acid cycle intermediate	↓ (7)		↑	Various metabolic functions including central metabolism, collagen synthesis, stem cell proliferation, and epigenetic regulation; leads to extended lifespan in mice [38]; in nutrient excess, promotes branched-chain amino acid catabolism [14]; stimulates autophagy [66]
NMMA (N-monomethyl-arginine)	Arginine derivative	↓ (20)		↓	Inhibitor of nitric oxide and potent vasoconstrictor [67]; exogenous administration leads to early satiety [68]

### Sensitivity analyses

By comparison, the change in glucose 2 h post-OGTT was not associated with incident CVD (P = 0.65) or mortality (P = 0.17) in multivariable-adjusted models in our study. To evaluate whether the information provided by Δmetabolites was complementary (i.e., additive) to traditional measures of dysglycemia and insulin resistance, we performed sensitivity analyses adjusting our multivariable models additionally for fasting glucose and HOMA-IR (Additional file 1: Table S4). We observed minimal attenuation of the effect estimates for Δmetabolites associated with CVD or mortality with these additional adjustments.

### Clinical and subclinical correlates of Δmetabolites

**Fig. 2:**
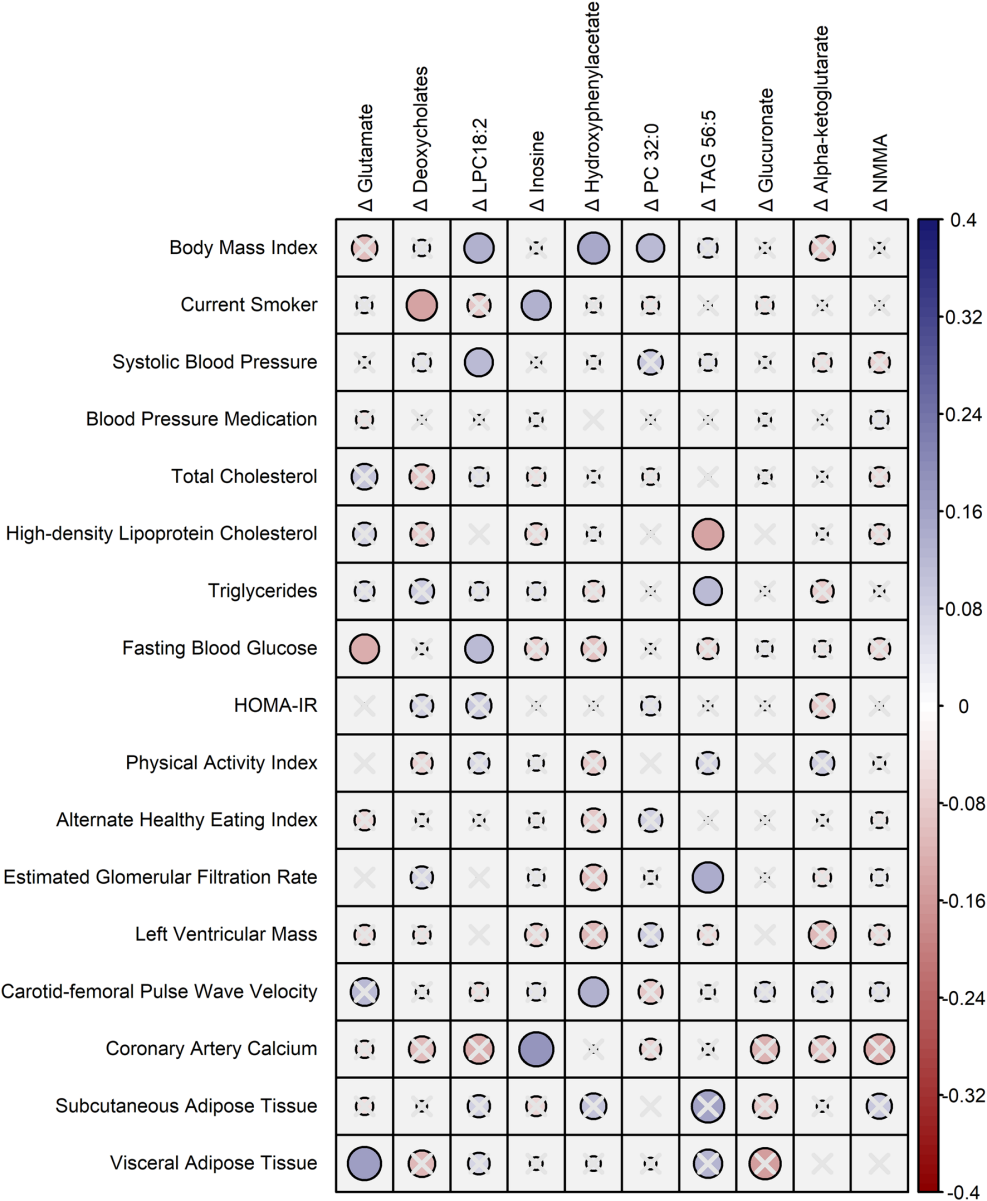
Clinical and subclinical correlates of Δmetabolites. Partial correlations (Spearman; adjusted for age, sex, and fasting metabolite level) of Δmetabolites (log2[post/pre] metabolite level) with clinical and subclinical measures are displayed. Carotid-femoral pulse wave velocity, coronary artery calcium, subcutaneous and visceral adipose tissue measures are from the 7th examination cycle (sample sizes shown in Table 1 footnote). All other measures were assessed contemporaneously with metabolites (5th examination cycle). Carotid-femoral pulse wave velocity was expressed as −1000/value, HOMA-iR was log-transformed, and coronary artery calcium was analyzed as the natural log of (value + 1). The area of each circle is proportional to the magnitude of the correlation coefficient and the circle color reflects the magnitude and direction of the correlation coefficient. Overlain “X” indicated that the correlation is not statistically significant at the P < 0.05 level

Next, we sought to further understand the clinical and subclinical correlates of changes in circulating metabolites that were related to CVD or mortality in risk factor-adjusted models (Fig. 2 and Additional file 1: Table S5). Overall, we observed modest correlations between Δmetabolites and traditional risk factors, health behaviors (e.g., physical activity, dietary quality), and subclinical disease measures, with variation across specific metabolites. For example, ΔTAG 56:5 (which was directly related to mortality) was correlated with lower HDL cholesterol and higher total triglycerides, eGFR, and subcutaneous adipose tissue volume, whereas Δhydroxyphenylacetate (also directly related to mortality) was correlated with higher body mass index, lower fasting blood glucose, and higher (adverse) CFPWV.

### Clinical implications

**Fig. 3:**
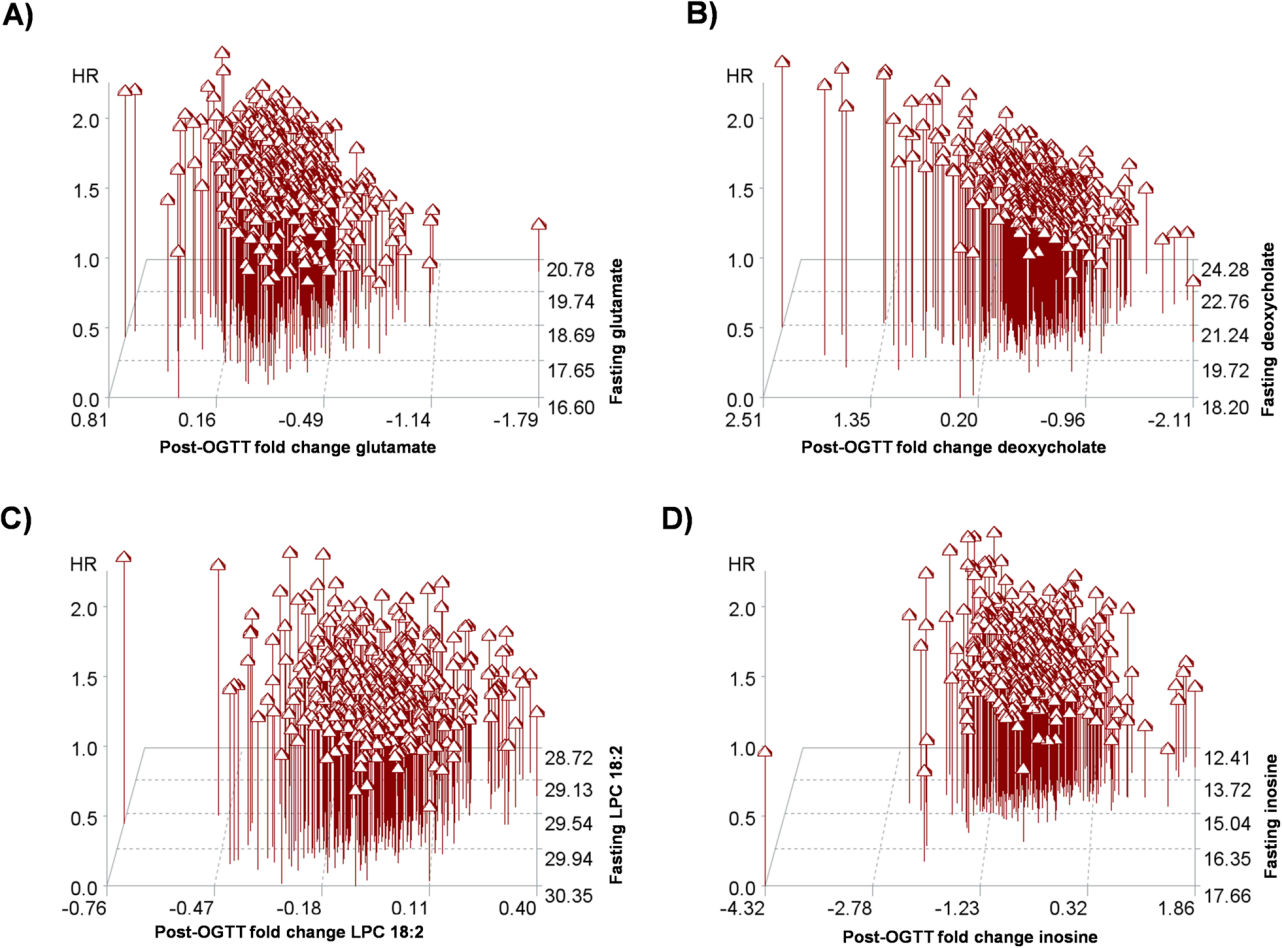
Clinical implications of Δmetabolite associations. Hazard ratios (adjusted for age, sex, fasting metabolite level, BMI, smoking, hypertension treatment, systolic blood pressure, total/HDL cholesterol) are represented by triangles and are plotted for each participant against the fasting metabolite level and the fold-change in response to an OGTT for the 4 metabolites with statistically significant associations of Δmetabolite and CVD (**A** glutamate; **B** deoxycholate; **C** LPC18:2, and **D** inosine)

Understanding the clinical implications (and directionality) of a higher change in Δmetabolites requires the integration of fasting metabolite levels and the direction of change after an OGTT. For example, a 1-SD higher change in glutamate from pre- to post-OGTT is associated with a 24% higher risk of CVD (in models adjusted for clinical risk factors), but on average, glutamate decreased by 20% with an OGTT in our sample. In Fig. 3A, we plot the pre-OGTT glutamate levels against their fold changes post-OGTT and estimated hazard ratios for each individual. As expected based on prior studies [39, 40], individuals with higher levels of fasting (i.e., pre-OGTT) glutamate had a higher predicted hazard of CVD. However, the post-OGTT fold-change provided incremental information on estimated CVD risk, with individuals with the highest fasting glutamate levels and increases in glutamate post-OGTT having a much higher risk of CVD when compared with those with higher resting levels but reduced glutamate post-OGTT. In addition, individuals with low fasting glutamate levels but increased levels post-OGTT demonstrated higher estimated CVD risk than would be expected by fasting measures alone. Similar trends were observed for other metabolite associations with CVD (Fig. 3).

## Discussion

We evaluated whether intraindividual changes in circulating metabolites in response to an OGTT would provide incremental information on the risk of CVD and mortality in community-dwelling individuals. We observed associations of metabolite changes in response to an OGTT with both CVD and mortality in models adjusted for fasting metabolite levels and clinical risk factors. Moreover, the fasting metabolite levels were not statistically significantly associated with CVD or mortality in comprehensively adjusted models in our sample, indicating that the OGTT-induced changes may provide prognostic information beyond fasting metabolites. Indeed, while changes in metabolites with an OGTT were only modestly correlated with clinical risk factors and subclinical disease measures, they helped to clarify risk assessments based on fasting metabolite levels. These findings demonstrate that the metabolomic response to a discrete metabolic perturbation (in this case, OGTT) may provide incremental information to baseline levels themselves.

In the search for new CVD biomarkers, many prior studies have related resting metabolite levels to cross-sectional and prospective cardiometabolic and CVD outcomes [10–20], but there is increasing interest in understanding how dynamic changes in metabolites within individuals may further uncover their underlying metabolic risk [21, 41, 42]. Prior studies have evaluated how responses of the circulating metabolome to an OGTT vary among individuals with prevalent risk factors. For example, Ho et al*.* reported on metabolite changes with an OGTT in this same sample, demonstrating significant changes in the majority of assayed metabolites [24]. These changes included metabolites reflecting reduced proteolysis and ketogenesis and increased glycolysis, as would be expected with glucose ingestion after a period of fasting, and were concordant with findings from Wang et al*.* reporting increased glycolysis intermediates, decreased branched-chain amino acids, ketone bodies, glycerol, and triglycerides in response to an OGTT [43]. There are also large shifts in different lipid species in response to an OGTT including reductions in circulating acylcarnitines (especially medium-chain), sphingolipids, and higher risk TAG and diacylglycerol species [13, 44]. Additionally, across several studies, many of these post-OGTT responses were blunted in individuals with insulin resistance compared to controls [13, 24, 43]. As insulin promotes glycolysis and suppresses lipolysis and proteolysis, these findings have been partially attributed to insulin resistance [44–46]. Moreover, Li-Gao et al*.* recently used genetic interrogation of the metabolomic response to an oral mixed meal tolerance test to identify novel loci regulating glycemic and lipid responses with links to diabetes and cardiometabolic disease [47]. However, the implications of metabolite changes in response to an OGTT on future health risk remain incompletely elucidated.

Our observed associations of metabolite changes in response to an OGTT with future CVD and mortality are noteworthy and open new avenues for further studies. We observed relations of OGTT-induced changes in metabolites with previous relations to cardiometabolic disease (e.g., glutamate, deoxycholate, TAGs, LPC 18:2) and metabolites with less defined links to cardiometabolic disease (e.g., inosine, hydroxyphenylacetate) to be associated with incident CVD and mortality. We suspect that our sample size may have limited our ability to detect statistically significant associations of some fasting metabolites. Yet, the observation of statistically significant associations of Δmetabolites with CVD and mortality risk is intriguing and raises the possibility that changes in metabolite levels in response to systemic perturbation might provide incremental information beyond baseline measures. In addition, intraindividual changes in a biomarker in response to stress/perturbation may have several advantages over single measurements as they can more readily account for measurement variability and confounding than can single timepoint measurements. Overall, our findings are consistent with the previous observation that blood glucose after a meal challenge is a better predictor of cardiovascular events in type 2 diabetes [48]. A glycemic load following a fast leads to a coordinated program of metabolic responses involving a shift from catabolic to anabolic processes. Limitations in rapidly switching metabolic states are an indicator of impaired “phenotypic flexibility”, an increasingly recognized indicator of metabolic health [23, 49, 50]. In this case, functional insulin resistance may play a role in blunted responses of circulating metabolites to a glycemic load and may partially underly these associations. While we did not observe significant correlations of key Δmetabolites with the measure of insulin resistance used in our study (HOMA-IR), insulin resistance can be challenging to assess and our findings therefore should not be interpreted as precluding a role for insulin resistance in impacting metabolic responses to an OGTT [51].

We observed changes in four metabolites to be associated with future CVD and six to be associated with future mortality in models adjusted for traditional clinical risk factors and fasting metabolite levels. Fasting glutamate levels have been linked with cardiometabolic disease implicating several putative mechanisms including gluconeogenesis, proteolysis, and inflammation [52, 53] (Table 4), and its levels usually fall after an OGTT. In our study, a blunted decrease (or even an increase) in glutamate following an OGTT was associated with a higher CVD risk. This finding is consistent with prior observations that blunting of metabolic changes with an OGTT is observed in individuals with higher cardiometabolic risk [24]. Similar blunting of OGTT-induced decreases in metabolites with established or putative links to cardiometabolic risk were observed for deoxycholate [54], and hydroxyphenylacetate [36, 37]. On the other hand, inosine, certain phosphatidylcholine (PC) species, lysoPC 18:2, and glucuronate share putative protective mechanisms in cardiometabolic disease and are observed to decrease with an OGTT. For these metabolites, less of a decrease in response to an OGTT was observed to be associated with a lower risk CVD or mortality. Taken together, these findings suggest that information about how an individual’s metabolism can adapt to a glucose challenge can provide incremental information regarding metabolic health, and conversely, metabolic risk.

Our study is one of the first to demonstrate long-term prospective associations of post-OGTT metabolite changes with relevant health outcomes. Nevertheless, there are several limitations of the present investigation. Our sample size was limited to individuals in whom OGTT was performed with metabolite profiling pre- and post-challenge. While this allowed us to evaluate associations with prospective outcomes and to compare the associations of resting metabolites and their delta, our statistical power was constrained, which likely explains why fasting levels of several metabolites previously linked with future CVD and mortality did not reach statistical significance in our sample. Due the limited sample size, we elected to determine statistical significance at a nominal threshold of P < 0.05 in this discovery effort; future studies in larger sample sizes with more racial diversity and inclusion of socioeconomic status assessment will be necessary to confirm these hypothesis-generating observations. Notably, our study sample included individuals with average BMI in the obese range and relatively high levels of other cardiometabolic risk factors. Therefore, whether our findings are applicable to individuals with more favorable cardiometabolic profiles is unknown. In addition, whether metabolite associations reflect causal mechanisms in CVD development vs. biomarkers of other biological processes (e.g., metabolic stress, inflammation) cannot be assessed by the current investigation and requires dedicated mechanistic studies. Metabolites were measured at two time points (pre- and post-OGTT); previous studies have shown that both shorter-term and longer-term changes in circulating analytes might provide additional information in response to a dietary challenge [55].

In conclusion, intraindividual changes in circulating metabolites in response to an OGTT are associated with CVD and mortality, are largely independent of traditional CVD risk factors, and provide incremental prognostic information beyond fasting metabolite levels. These findings indicate that metabolic responses to an OGTT may be able to identify individuals at increased risk before developing overt traditional risk factors, providing new opportunities for targeting earlier, and even “primordial,” prevention of CVD and cardiometabolic disease. Further studies are necessary to confirm these findings in larger samples with more diverse populations in which rigorous assessment for risk prediction can be performed and to assess whether other systemic responses to discrete perturbations may also augment traditional risk prediction methods.

## Supplementary Information

**Additional file 1: Table S1.** Characteristics of the study sample by outcome group. **Table S2.** Comparison of the study subsample to the full FHS Generation 2. **Table S3.** Changes in metabolites from pre- to post-OGTT. **Table S4a.** Association of Δmetabolites with incident CVD with additional adjustment for fasting blood glucose and HOMA-IR. **Table S4b.** Association of Δmetabolites with mortality with additional adjustment for fasting blood glucose and HOMA-IR. **Table S5.** Spearman Rank Correlation partial coefficients adjusted for age, sex, and fasting metabolite level.

## Data Availability

The data supporting the findings of this study will be made available on reasonable request. Framingham Heart Study data are publicly available and can be accessed through the National Institutes of Health database of genotypes and phenotypes (https://www.ncbi.nlm.nih.gov/gap/).

## Abbreviations

CVD: Cardiovascular disease
OGTT: Oral glucose tolerance test
FHS: Framingham Heart Study
LC–MS: Liquid chromatography–mass spectrometry
HOMA-IR: Homeostatic Model of Insulin Resistance
CFPWV: Carotid-femoral pulse wave velocity
FDR: False discovery rate
Δmetabolite: Pre- to post-OGTT change in metabolite level
TAG: Triacylglycerol
LPC: Lysophosphatidylcholine
NMMA: N-monomethyl arginine
