# Supplementary material for: Response of circulating metabolites to an oral glucose challenge and risk of cardiovascular disease and mortality in the community
Source: Cardiovasc Diabetol. 2022 Oct 15;21:213. doi: 10.1186/s12933-022-01647-w (PMC9568897; doi:10.1186/s12933-022-01647-w)
Supplement: Supplementary file 1 — Additional file 1: Table S1. Characteristics of the study sample by outcome group. Table S2. Comparison of the study subsample to the full FHS Generation 2. Table S3. Changes in metabolites from pre- to post-OGTT. Table S4a. Association of Δmetabolites with incident CVD with additional adjustment for fasting blood glucose and HOMA-IR. Table S4b. Association of Δmetabolites with mortality with additional adjustment for fasting blood glucose and HOMA-IR. Table S5. Spearman Rank Correlation partial coefficients adjusted for age, sex, and fasting metabolite level. [file 12933_2022_1647_MOESM1_ESM.pdf]

SUPPLEMENTAL MATERIAL

Supplemental Table 1. Characteristics of the study sample by outcome group

Characteristic	No CVD (n=229)	CVD (n=132)	P-value	No death (n=217)	Death (n=144)	p
Age, years	55±8	59±9	<0.001	53±7	62±8	<0.001
Women	96 (41%)	55 (42%)	1.00	96 (44%)	55 (38%)	0.302
Body mass index, kg/m^2	29.8±5.3	31.0±5.1	0.034	30.2±5.2	30.3±5.4	0.849
Systolic blood pressure, mm Hg	132±17	136±19	0.027	131±17	137±18	0.002
Hypertension treatment	54 (23%)	49 (38%)	0.007	50 (23%)	53 (37%)	0.006
Current smoking	29 (13%)	29 (22%)	0.03	28 (13%)	30 (21%)	0.062
Total cholesterol, mg/dl	210.3±36.5	211.8±36.1	0.693	210.2±36.3	211.8±36.5	0.691
HDL cholesterol, mg/dl	46.5±13.5	43.1±11.5	0.016	45.0±12.3	45.7±13.8	0.624
Triglycerides, mg/dl	167±111	176±93	0.404	172±107	167±102	0.659
Fasting blood glucose, mg/dl	105±8	105±9	0.648	104±8	106±9	0.015
HOMA-IR	1.23±0.47	1.43±0.57	<0.001	1.25±0.45	1.37±0.60	0.033
Physical activity index	35±8	33±7	0.013	35±8	34±7	0.435
Alternate healthy eating index	53±12	51±11	0.221	53±12	51±12	0.214
Estimated glomerular filtration rate, ml/min/1.73 m^2	90±18	88±21	0.381	93±17	84±22	<0.001
Left ventricular mass, grams	171±37	186±43	0.004	173±37	183±44	0.052
Carotid femoral pulse wave velocity, m/s	10.0 (8.2-11.6)	10.9 (9.5-13.9)	<0.001	9.7 (8.1-11.2)	11.2 (9.9-13.7)	<0.001
Coronary artery calcium, Hounsfield units	69 (0-348)	479 (118-1369)	<0.001	79 (0-348)	676 (131-1779)	<0.001
Subcutaneous adipose tissue, cm^3	3133±1324	3331±1362	0.399	3298±1410	3025±1164	0.262
Visceral adipose tissue, cm^3	2681±1050	3126±1142	0.021	2647±975	3277±1239	0.001

Data are presented as mean±SD, median (25th-75th percentile), or N(% of total). Values for those with outcome vs. without are compared using t-test for continuous data and chi-squared tests for categorical data.

Supplemental Table 2. Comparison of the study subsample to the full FHS Generation 2 sample

Characteristic	Study sample (N=361)	Generation 2 sample (N=3799)	P-value
Age, years	56±9	55±10	0.014
Women	151 (42%)	2007 (53%)	<0.001
Body mass index, kg/m2	30.2±5.3	27.4±5.0	<0.001
Systolic blood pressure, mm Hg	133±18	126±19	<0.001
Hypertension treatment	103 (29%)	731 (19%)	<0.001
Current smoking	58 (16%)	746 (20%)	0.114
Total cholesterol, mg/dL	210.8±36.3	204.8±36.8	0.003
HDL cholesterol, mg/dL	45.3±12.9	49.8±15.2	<0.001

Data in table are mean ± SD or N(% of total). Sample characteristics were compared with t-tests for continuous data and chi-squared tests for categorical data.

Supplemental Table 3. Changes in metabolites from pre- to post-OGTT

Metabolite	Geometric mean fold change	Mean log2 fold change	SD log2 fold change	P-value	FDR P-value
citrulline	0.606	-0.723	0.213	6.75E-209	1.42E-206
isoleucine	0.623	-0.682	0.208	4.41E-204	4.65E-202
leucine	0.639	-0.647	0.210	1.90E-194	1.34E-192
tyrosine	0.712	-0.491	0.197	3.74E-164	1.97E-162
threonine	0.764	-0.388	0.157	1.29E-162	5.45E-161
valine	0.791	-0.338	0.139	4.34E-160	1.53E-158
beta_hydroxybutyrate	0.222	-2.173	1.040	7.63E-140	2.30E-138
hydroxyproline	0.732	-0.451	0.223	2.89E-135	7.62E-134
methionine	0.694	-0.526	0.271	3.91E-130	9.17E-129
hippurate	4.630	2.211	1.148	4.52E-129	9.54E-128
phenylalanine	0.771	-0.376	0.196	3.56E-128	6.84E-127
proline	0.874	-0.195	0.127	2.79E-101	4.91E-100
serine	0.786	-0.347	0.239	1.44E-94	2.34E-93
asparagine	0.735	-0.445	0.350	1.19E-80	1.80E-79
pyridoxate	0.821	-0.285	0.231	9.22E-78	1.30E-76
xanthurenate	0.678	-0.560	0.451	1.67E-77	2.21E-76
histidine	0.847	-0.240	0.200	4.65E-75	5.77E-74
kynurenic	0.678	-0.560	0.469	1.67E-74	1.95E-73
lysine	0.844	-0.244	0.208	6.51E-73	7.23E-72
tryptophan	0.851	-0.232	0.201	2.94E-71	3.10E-70
aminoisobutyric	0.753	-0.410	0.357	1.40E-70	1.41E-69
taurine	0.874	-0.195	0.177	4.92E-67	4.72E-66
aminoadipate	0.780	-0.358	0.329	6.49E-66	5.96E-65
PCA36_4	0.926	-0.111	0.108	2.14E-61	1.88E-60
ADMA_SDMA	0.860	-0.217	0.213	2.24E-60	1.89E-59
ornithine	0.777	-0.364	0.359	1.05E-59	8.52E-59
serotonin	0.429	-1.221	1.199	1.26E-58	9.83E-58
alpha_hydroxybutyrate	0.854	-0.228	0.251	1.86E-51	1.40E-50
xanthine	0.595	-0.749	0.828	7.89E-51	5.74E-50
aspartate	0.765	-0.387	0.431	1.64E-50	1.15E-49
glycine	0.868	-0.205	0.238	3.94E-47	2.68E-46
orotate	0.789	-0.342	0.411	4.42E-45	2.91E-44
inosine	0.670	-0.577	0.706	1.71E-43	1.09E-42
propionate	0.687	-0.542	0.604	1.12E-40	6.98E-40
hypoxanthine	0.461	-1.118	1.347	5.23E-40	3.15E-39
NMMA	0.873	-0.196	0.255	8.43E-40	4.94E-39
gentisate	0.711	-0.492	0.673	5.03E-37	2.87E-36
cystathionine	0.803	-0.317	0.460	8.44E-34	4.69E-33
xanthosine	0.844	-0.245	0.357	1.45E-33	7.82E-33
LPE16_0	1.108	0.148	0.220	1.06E-32	5.61E-32
niacinamide	0.763	-0.391	0.580	1.44E-32	7.43E-32

hydroxyphenylacetate	0.882	-0.181	0.268	1.83E-32	9.21E-32
glutamate	0.872	-0.197	0.297	1.11E-31	5.44E-31
GDP	0.613	-0.705	1.042	2.18E-31	1.05E-30
phosphoglycerate	1.483	0.569	0.873	6.54E-31	3.07E-30
PC32_2	0.946	-0.080	0.123	2.27E-30	1.04E-29
pantothenate	0.911	-0.135	0.215	2.36E-29	1.06E-28
UDP	0.761	-0.394	0.617	8.65E-29	3.80E-28
TAG56_6	1.066	0.092	0.148	1.17E-28	5.04E-28
PC34_3	0.962	-0.056	0.091	4.18E-28	1.76E-27
alpha_glycerophosphate	1.351	0.434	0.707	4.70E-28	1.95E-27
arginine	0.807	-0.310	0.510	1.31E-27	5.31E-27
uridine	0.911	-0.135	0.225	4.40E-27	1.75E-26
lactate	1.139	0.188	0.321	7.19E-26	2.81E-25
oxalate	0.890	-0.168	0.303	1.45E-23	5.57E-23
GMP	0.689	-0.537	0.945	1.02E-22	3.83E-22
PC32_0	0.975	-0.036	0.069	4.52E-21	1.67E-20
fumarate_maleate	0.893	-0.163	0.318	6.05E-21	2.20E-20
alanine	0.935	-0.097	0.193	2.69E-20	9.60E-20
TAG56_8	1.084	0.117	0.239	2.55E-19	8.97E-19
indole_propionate	0.886	-0.174	0.359	6.91E-19	2.39E-18
aconitate	0.903	-0.147	0.308	1.47E-18	5.00E-18
PC34_4	0.949	-0.075	0.159	2.53E-18	8.47E-18
TAG56_4	1.061	0.086	0.181	2.86E-18	9.44E-18
deoxycholates	0.824	-0.280	0.595	3.28E-18	1.07E-17
UMP	0.798	-0.325	0.668	4.89E-18	1.56E-17
TAG56_7	1.061	0.085	0.189	6.13E-17	1.93E-16
dimethylglycine	0.947	-0.078	0.174	7.39E-17	2.29E-16
PC36_2	0.987	-0.019	0.042	1.65E-16	5.05E-16
isocitrate	0.926	-0.111	0.258	1.31E-15	3.94E-15
AMP	0.722	-0.470	1.098	1.59E-15	4.73E-15
TAG46_2	0.928	-0.108	0.260	1.01E-14	2.95E-14
TAG58_8	1.080	0.111	0.273	2.49E-14	7.19E-14
TAG58_10	1.077	0.107	0.264	2.86E-14	8.15E-14
quinolinate	0.936	-0.096	0.238	4.01E-14	1.13E-13
carnitine	1.035	0.049	0.121	4.84E-14	1.34E-13
SM14_0	0.970	-0.044	0.109	5.76E-14	1.58E-13
TAG58_9	1.070	0.098	0.245	6.47E-14	1.75E-13
tauro_deoxycholates	1.409	0.495	1.244	9.69E-14	2.59E-13
TAG44_1	0.914	-0.130	0.338	4.88E-13	1.29E-12
LPE20_4	0.929	-0.107	0.279	5.14E-13	1.34E-12
methyladipate_pimelate	0.932	-0.102	0.266	5.36E-13	1.38E-12
glycerophosphocholine	1.156	0.209	0.550	1.06E-12	2.70E-12
PC34_2	0.990	-0.014	0.037	1.25E-12	3.13E-12
PC32_1	0.977	-0.034	0.089	1.42E-12	3.53E-12
ribose_ribulose_p	0.860	-0.217	0.577	1.73E-12	4.23E-12

glutamine	0.908	-0.140	0.373	1.94E-12	4.70E-12
glyco_deoxycholates	1.352	0.435	1.180	3.95E-12	9.45E-12
trimethylamine	0.917	-0.125	0.339	3.99E-12	9.45E-12
creatine	1.051	0.072	0.197	6.77E-12	1.59E-11
HIAA	0.868	-0.205	0.570	1.20E-11	2.78E-11
TAG48_1	0.959	-0.060	0.169	3.09E-11	7.09E-11
TAG58_12	1.069	0.096	0.273	3.24E-11	7.35E-11
betaine	1.027	0.038	0.110	5.48E-11	1.23E-10
sorbitol	0.935	-0.097	0.278	5.70E-11	1.27E-10
ADP	0.751	-0.413	1.205	9.65E-11	2.12E-10
TAG46_1	0.939	-0.091	0.272	2.40E-10	5.21E-10
urate	0.969	-0.046	0.138	2.56E-10	5.52E-10
fruc_gluc_galac	1.045	0.064	0.195	6.20E-10	1.32E-09
TAG58_11	1.050	0.070	0.219	1.27E-09	2.67E-09
PEP	1.110	0.150	0.470	1.56E-09	3.27E-09
SM16_1	0.979	-0.031	0.099	3.36E-09	6.95E-09
phosphocreatine	1.105	0.144	0.468	5.24E-09	1.07E-08
TAG48_3	0.955	-0.067	0.223	1.21E-08	2.45E-08
glucuronate	0.932	-0.101	0.338	1.44E-08	2.89E-08
TAG56_5	1.060	0.084	0.285	1.95E-08	3.89E-08
PC36_1	0.989	-0.016	0.055	3.89E-08	7.68E-08
inositol	1.059	0.083	0.282	4.54E-08	8.87E-08
PC38_6	1.017	0.025	0.088	9.67E-08	1.87E-07
LPE18_1	0.957	-0.063	0.229	1.36E-07	2.62E-07
taurocholate	1.266	0.340	1.219	1.55E-07	2.94E-07
hydroxyglutarate	0.938	-0.093	0.355	5.02E-07	9.45E-07
TAG48_2	0.961	-0.058	0.223	6.65E-07	1.24E-06
LPC20_3	0.946	-0.080	0.311	9.77E-07	1.81E-06
CE18_3	0.975	-0.036	0.142	1.01E-06	1.85E-06
non_carnitine	1.061	0.086	0.346	1.84E-06	3.35E-06
glycocholate	1.166	0.222	0.911	2.92E-06	5.27E-06
suberate	0.956	-0.065	0.270	3.75E-06	6.70E-06
SM16_0	0.992	-0.011	0.047	3.98E-06	7.06E-06
PC38_5	1.010	0.014	0.057	4.33E-06	7.62E-06
choline	1.028	0.040	0.170	5.89E-06	1.03E-05
PC40_6	1.015	0.022	0.091	6.13E-06	1.06E-05
LPE18_2	0.956	-0.065	0.280	9.02E-06	1.55E-05
FMN	0.897	-0.157	0.640	1.67E-05	2.83E-05
adipate	0.951	-0.072	0.326	2.29E-05	3.87E-05
LPC18_2	0.975	-0.036	0.166	3.76E-05	6.29E-05
PC36_3	0.994	-0.009	0.043	6.16E-05	1.02E-04
SM24_0	0.987	-0.019	0.091	6.48E-05	1.07E-04
thiamine	0.865	-0.210	1.016	7.16E-05	1.17E-04
sebacate	0.961	-0.057	0.276	7.93E-05	1.29E-04
SM22_0	0.991	-0.013	0.066	1.21E-04	1.96E-04

TAG60_12	1.053	0.075	0.382	1.56E-04	2.49E-04
anthranilic	0.947	-0.079	0.410	2.22E-04	3.51E-04
TAG54_6	1.026	0.037	0.194	2.47E-04	3.89E-04
LPC22_6	0.964	-0.053	0.278	2.51E-04	3.92E-04
PC38_3	1.010	0.014	0.075	2.96E-04	4.59E-04
F1P_F6P_G1P_G6P	0.949	-0.075	0.411	4.35E-04	6.69E-04
TAG48_4	0.968	-0.047	0.264	5.62E-04	8.59E-04
TAG50_3	0.979	-0.030	0.171	6.14E-04	9.30E-04
allantoin	0.947	-0.078	0.440	6.17E-04	9.30E-04
TAG54_9	0.972	-0.041	0.238	8.00E-04	1.20E-03
LPC18_1	0.983	-0.025	0.149	1.04E-03	1.54E-03
TAG54_3	1.015	0.022	0.128	1.04E-03	1.54E-03
SM18_1	0.990	-0.015	0.086	1.07E-03	1.57E-03
TAG54_5	1.017	0.024	0.143	1.11E-03	1.61E-03
spermine	1.060	0.084	0.500	1.18E-03	1.71E-03
PC34_1	0.996	-0.006	0.040	1.82E-03	2.61E-03
LPC20_4	0.976	-0.035	0.221	1.97E-03	2.80E-03
GABA	0.928	-0.108	0.678	2.07E-03	2.93E-03
indoxylsulfate	0.961	-0.057	0.356	2.13E-03	3.00E-03
TAG54_4	1.011	0.016	0.102	2.20E-03	3.08E-03
DAG36_1	0.964	-0.053	0.335	2.29E-03	3.18E-03
CE20_5	0.981	-0.027	0.172	2.44E-03	3.37E-03
TAG54_2	0.980	-0.029	0.185	2.58E-03	3.54E-03
sucrose	1.107	0.147	0.947	2.70E-03	3.68E-03
CE18_2	0.991	-0.013	0.083	3.35E-03	4.53E-03
TAG50_4	0.977	-0.033	0.226	5.02E-03	6.75E-03
TAG50_2	0.990	-0.015	0.107	6.56E-03	8.76E-03
CE20_4	0.988	-0.017	0.128	0.012	0.015
LPC16_1	1.019	0.027	0.210	0.012	0.016
CE16_1	0.986	-0.021	0.168	0.015	0.020
LPC20_5	0.977	-0.033	0.264	0.015	0.020
CE16_0	0.990	-0.015	0.122	0.018	0.024
alpha_ketoglutarate	0.950	-0.074	0.601	0.023	0.029
TAG52_1	0.986	-0.021	0.182	0.026	0.033
CE18_1	0.991	-0.013	0.113	0.031	0.040
SM24_1	0.995	-0.007	0.063	0.036	0.046
LPC16_0	1.006	0.009	0.084	0.039	0.049
SM18_0	0.994	-0.008	0.081	0.044	0.055
succinate_methylmalonate	0.979	-0.030	0.286	0.045	0.056
creatinine	0.992	-0.012	0.118	0.045	0.056
UDP_galactose_glucose	0.940	-0.090	0.902	0.054	0.067
malate	0.981	-0.028	0.284	0.058	0.071
thyroxine	0.979	-0.030	0.308	0.059	0.071
citrate	0.987	-0.019	0.201	0.061	0.073
adenosylhomocysteine	0.954	-0.068	0.707	0.061	0.073

TAG56_9	1.013	0.019	0.205	0.076	0.090
TAG54_7	1.013	0.018	0.209	0.09	0.11
SM18_2	0.991	-0.013	0.158	0.10	0.12
TAG52_3	1.004	0.006	0.072	0.10	0.12
DAG36_2	0.986	-0.020	0.254	0.12	0.14
TAG50_5	0.988	-0.018	0.254	0.16	0.19
pyruvate	1.042	0.059	0.780	0.18	0.21
LPC14_0	1.013	0.018	0.266	0.19	0.22
cAMP	0.970	-0.044	0.624	0.19	0.22
CE14_0	0.979	-0.031	0.466	0.20	0.23
LPC18_0	1.006	0.008	0.124	0.23	0.26
TAG52_4	1.005	0.007	0.116	0.25	0.28
CE22_6	0.990	-0.015	0.273	0.29	0.33
DAG34_1	0.993	-0.010	0.190	0.33	0.36
TAG56_3	0.990	-0.014	0.274	0.34	0.37
kynurenine	0.995	-0.007	0.168	0.39	0.43
TAG52_6	0.994	-0.009	0.199	0.40	0.44
TAG52_2	1.003	0.005	0.121	0.44	0.48
TAG48_0	1.011	0.016	0.409	0.46	0.50
SM22_1	1.001	0.002	0.070	0.49	0.53
DAG34_2	1.006	0.008	0.250	0.51	0.55
PC38_2	0.998	-0.003	0.097	0.51	0.55
lactose	0.985	-0.022	0.745	0.57	0.60
LPE18_0	1.004	0.006	0.225	0.63	0.66
CE20_3	0.997	-0.005	0.200	0.63	0.66
TAG54_8	0.997	-0.005	0.209	0.65	0.68
carbamoylalanine	0.997	-0.005	0.250	0.69	0.72
TAG52_5	0.997	-0.004	0.201	0.70	0.73
PC38_4	1.001	0.001	0.056	0.72	0.74
TAG50_0	0.997	-0.005	0.315	0.75	0.77
PCB36_4	0.999	-0.001	0.052	0.76	0.77
TAG56_10	1.002	0.003	0.253	0.83	0.84
CE18_0	1.003	0.004	0.336	0.83	0.84
TAG54_10	1.002	0.003	0.306	0.86	0.87
LPE22_6	1.001	0.001	0.169	0.87	0.87

P-values were calculated comparing the observed log2 fold change to 0 (reflecting no change)
Geometric means were calculated as 2^(mean log2 fold change)

Supplemental Table 4a. Association of Δmetabolites with incident CVD with additional adjustment for fasting blood glucose and HOMA-IR

	With fasting blood glucose			With log(HOMA-IR)		
**Metabolite**	**HR**	**(95% CI)**	**P-value**	**HR**	**(95% CI)**	**P-value**
Glutamate	1.23	(1.02-1.49)	0.03	1.23	(1.02-1.48)	0.04
Deoxycholate	1.26	(1.02-1.55)	0.03	1.22	(0.99-1.51)	0.06
LPC 18:2	0.80	(0.65-0.99)	0.04	0.78	(0.64-0.96)	0.02
Inosine	0.81	(0.66-0.99)	0.04	0.83	(0.68-1.02)	0.07

Supplemental Table 4b. Association of Δmetabolites with mortality with additional adjustment for fasting blood glucose and HOMA-IR

	With fasting blood glucose			With log(HOMA-IR)		
**Metabolite**	**HR**	**(95% CI)**	**P-value**	**HR**	**(95% CI)**	**P-value**
Hydroxyphenylacetate	1.29	(1.06-1.57)	0.01	1.30	(1.07-1.58)	0.009
PC 32:0	0.81	(0.69-0.95)	0.01	0.82	(0.70-0.96)	0.02
TAG 56:5:	1.28	(1.04-1.57)	0.02	1.29	(1.05-1.59)	0.02
Glucuronate	0.84	(0.72-0.98)	0.03	0.85	(0.73-0.99)	0.04
Alpha-ketoglutarate	1.22	(1.01-1.46)	0.04	1.22	(1.02-1.46)	0.03
NMMA	0.83	(0.69-0.99)	0.04	0.84	(0.71-1.01)	0.06

Δ Metabolite is the log2 fold-change from pre- to post-OGTT and baseline metabolites were log-transformed. Baseline and change metabolite values were standardized (mean 0 and SD 1).
The hazard ratio (HR) represents the relative hazard for a 1-SD higher log2 fold-change in the metabolite. Models are adjusted for age, sex, BMI, smoking, hypertension treatment, systolic blood pressure, total/HDL cholesterol, fasting metabolite levels, and the variable listed in the column heading.

Supplemental Table 5. Spearman Rank Correlation partial coefficients adjusted for age, sex, and fasting metabolite level

Clinical Characteristic	Glutamate	Deoxycholates	LPC18:2	Inosine	Hydroxy-phenylacetate	PC32:0	TAG 56:5	Glucoronate	α-keto-gluturate	NMMA
Body mass index	-0.10	0.04	0.13*	-0.02	0.15*	0.12*	0.06	-0.02	-0.10	-0.02
Current smoking	0.04	-0.14*	-0.08	0.13*	-0.03	-0.05	0.01	-0.05	-0.01	-0.01
Systolic blood pressure	0.02	0.05	0.12*	0.02	-0.03	0.09	0.04	-0.02	-0.05	-0.07
Blood pressure medication	-0.05	-0.01	-0.01	0.03	0.00	0.01	0.01	0.02	0.01	0.05
Total cholesterol	0.10	-0.09	0.06	-0.05	-0.02	-0.04	0.00	-0.03	-0.02	-0.06
HDL cholesterol	0.08	-0.08	0.00	-0.07	0.03	0.00	-0.14*	0.00	0.02	-0.06
Triglycerides	0.06	0.09	0.05	0.05	-0.07	0.01	0.12*	0.01	-0.08	-0.02
Fasting blood glucose	-0.13*	0.02	0.12*	-0.08	-0.09	0.01	-0.07	0.04	-0.04	-0.07
HOMA-IR	0.00	0.08	0.10	-0.01	-0.01	0.06	0.01	0.01	-0.09	-0.01
Physical activity index	0.00	-0.07	0.07	0.04	-0.09	0.00	0.08	0.00	0.09	-0.02
Alternate healthy eating index	-0.06	-0.02	0.02	-0.03	-0.09	0.08	-0.01	-0.01	-0.02	-0.04
Estimated glomerular filtration rate	0.00	0.08	0.00	0.04	-0.10	-0.03	0.14*	0.01	-0.05	0.04
Left ventricular mass	-0.06	-0.05	0.00	-0.08	-0.11	0.09	-0.06	0.00	-0.11	-0.07
Carotid femoral pulse wave velocity	0.12	0.03	-0.05	0.05	0.13*	-0.09	0.03	0.06	0.07	0.06
Coronary artery calcium	-0.05	-0.11	-0.13	0.18*	0.01	-0.07	0.02	-0.12	-0.10	-0.14
Subcutaneous adipose tissue	-0.05	0.02	0.08	-0.06	0.10	0.00	0.16	-0.09	0.02	0.10
Visceral adipose tissue	0.17*	-0.11	0.07	-0.03	0.03	-0.02	0.12	-0.15	0.00	0.00

*Represents correlation coefficients with $P<0.05$

Carotid-femoral pulse wave velocity, coronary artery calcium, subcutaneous and visceral adipose tissue measures are from the 7th examination cycle (sample sizes shown in **Table 1** footnote)

Carotid femoral pulse wave velocity was transformed (-1000/CFPWV) for analysis, coronary artery calcium was analyzed as log(1+coronary artery calcium score), and HOMA-IR was log-transformed.
